# Effect of a 16-week Bikram yoga program on heart rate variability and associated cardiovascular disease risk factors in stressed and sedentary adults: A randomized controlled trial

**DOI:** 10.1186/s12906-017-1740-1

**Published:** 2017-04-21

**Authors:** Zoe L. Hewett, Kate L. Pumpa, Caroline A. Smith, Paul P. Fahey, Birinder S. Cheema

**Affiliations:** 1 School of Science and Health, Western Sydney University, Locked Bag 1797, Penrith, NSW 2751 Australia; 2 National Institute of Complementary Medicine, Western Sydney University, Penrith, NSW 2751 Australia; 3 Research Institute for Sport and Exercise, University of Canberra, ACT, Canberra, 2617 Australia

**Keywords:** *Hatha* yoga, obesity, overweight, inactivity, health, metabolic disease, physiological stress

## Abstract

**Background:**

Chronic activation of the stress-response can contribute to cardiovascular disease risk, particularly in sedentary individuals. This study investigated the effect of a Bikram yoga intervention on the high frequency power component of heart rate variability (HRV) and associated cardiovascular disease (CVD) risk factors (i.e. additional domains of HRV, hemodynamic, hematologic, anthropometric and body composition outcome measures) in stressed and sedentary adults.

**Methods:**

Eligible adults were randomized to an experimental group (*n* = 29) or a no treatment control group (*n* = 34). Experimental group participants were instructed to attend three to five supervised Bikram yoga classes per week for 16 weeks at local studios. Outcome measures were assessed at baseline (week 0) and completion (week 17).

**Results:**

Sixty-three adults (37.2 ± 10.8 years, 79% women) were included in the intention-to-treat analysis. The experimental group attended 27 ± 18 classes. Analyses of covariance revealed no significant change in the high-frequency component of HRV (*p* = 0.912, partial *η* ^2^ = 0.000) or in any secondary outcome measure between groups over time. However, regression analyses revealed that higher attendance in the experimental group was associated with significant reductions in diastolic blood pressure (*p* = 0.039; partial *η* ^2^ = 0.154), body fat percentage (*p* = 0.001, partial *η* ^2^ = 0.379), fat mass (*p* = 0.003, partial *η* ^2^ = 0.294) and body mass index (*p* = 0.05, partial *η* ^2^ = 0.139).

**Conclusions:**

A 16-week Bikram yoga program did not increase the high frequency power component of HRV or any other CVD risk factors investigated. As revealed by post hoc analyses, low adherence likely contributed to the null effects. Future studies are required to address barriers to adherence to better elucidate the dose-response effects of Bikram yoga practice as a medium to lower stress-related CVD risk.

**Trial registration:**

Retrospectively registered with Australia New Zealand Clinical Trials Registry ACTRN12616000867493. Registered 04 July 2016.

## Background

Chronic psychological stress is associated with increased risk of cardiovascular disease (CVD) and associated mortality [1–3]. During stress, the sympathetic nervous system (SNS) mediates neuroendocrine changes via the hypothalamic-pituitary-adrenal (HPA) axis [4, 5]. This ‘fight-or-flight’ response includes the release of stress hormones (e.g. cortisol, aldosterone, epinephrine), which in turn increase heart rate, blood pressure, and blood lipid and glucose concentrations, preparing the body for physical exertion. Chronic SNS activity, via this mechanism, can contribute to atherosclerosis and CVD, particularly in individuals who are physically inactive (sedentary) [6, 7].

Heart rate variability (HRV) is the instantaneous variation in heart rhythm due to autonomic nervous system (ANS) influences on the sinoatrial node. Low HRV is associated with a reduced capacity to adjust to environmental demands [8, 9] and increased CVD and mortality [1, 9, 10]. Moreover, low HRV is consistently noted in individuals who are sedentary [11], overweight-obese [12, 13] and psychologically stressed [3]. Specific components of HRV can denote the relative input of each branch of the ANS. Vagal activity is reflected in the high frequency (HF) spectral power component of HRV [14].

*Hatha* yoga is a branch of yoga originating from India that emphasizes the performance of physical postures (*asanas*) [15]. Several studies have reported that *hatha* yoga can reduce perceived stress [16] and salivary cortisol, a main effector of the SNS and HPA axis pathways [17–19], as well as improve cardiometabolic health [20]. Studies have also reported that a single session of *hatha* yoga can acutely increase the HF power component of HRV [21–24]. However, the chronic effects of *hatha* yoga training on HRV remain inconclusive due to a lack of robust clinical trials [25].

Bikram yoga is a specific system of *hatha* yoga that incorporates a 90-min, unchanging sequence of *asanas* performed in a heated environment (40.6 °C, 40% humidity) [26]. A randomized controlled trial (RCT) of Bikram yoga reported reduced reactivity to stress (cortisol) in women at risk for obesity-related illnesses [27], while uncontrolled trials have shown that 8-weeks of Bikram yoga can reduce perceived stress in apparently healthy adults [28], reduce blood lipids (i.e. total and low-density lipoprotein cholesterol) and arterial stiffness in healthy adults [29] and improve glucose tolerance, body mass and body mass index (BMI) in obese adults [30]. The heated environment is a cornerstone feature of Bikram yoga and may aid in its effectiveness in abating CVD risk factors. For example, recent prospective data indicates that more frequent sauna bathing is associated with reduced risk of sudden cardiac death, coronary heart disease, and all-cause mortality in males [31]. Further, preliminary evidence suggests that thermal exposure (sauna bathing and spa treatment) may lead to increased resting HRV in healthy subjects and athletes [32, 33].

To date, no study has investigated the effect of Bikram yoga on any HRV outcomes [34]. Therefore, the purpose of this study was to investigate the effect of a 16-week Bikram yoga intervention on the HF power component of HRV and associated CVD risk factors in a population of stressed and sedentary adults. We hypothesized that participants randomized to the intervention would significantly increase the HF power component and experience significant adaptation of associated CVD risk factors, including additional HRV measures (time and frequency domains) and a range of hemodynamic, hematologic, anthropometric and body composition outcomes compared to a no treatment control.

## Methods

### Study design

This 16-week, parallel-arm RCT compared the outcomes of participants randomized to an experimental group (Bikram yoga) or a no-treatment control group. Primary and secondary outcomes were collected prior to and following the intervention period at weeks 0 and 17, respectively. The Western Sydney University (H10549) and University of Canberra Human Research Ethics Committees (H10549-14/009174) approved all procedures and written informed consent was obtained from all participants.

### Participants and recruitment

*Eligibility criteria*: (1) Adult (>18 years); (2) sedentary (i.e. <150 min of moderate-intensity exercise per week [35] for greater than 6 months); (3) a score > 14 on the stress component of the Depression, Anxiety and Stress Scale (DASS-21) [36] indicating mild stress; (4) no diagnosed chronic diseases; (5) no acute or chronic medical conditions which would make Bikram yoga potentially hazardous (i.e. pregnancy) or primary outcome difficult to assess (i.e. pacemaker influences on HRV); (6) able to attend three to five Bikram yoga classes per week for 16 weeks; (7) cognition and English language sufficient to understand research procedures and provide informed consent; (8) no participation in Bikram yoga in the past 6 months. Original inclusion criteria included waist circumference ≥ 94 cm for men and ≥80 cm for women but was removed due to low rate of recruitment.

Participants were recruited between August 2014 and September 2015 in the Australian Capital Territory (ACT) using flyers (posted at local community dwellings, and shared via social media) and word of mouth referral. Individuals who contacted the principal investigator and expressed interest were assessed for eligibility using a standardized screening process and questionnaires administered via email and telephone interview. An individual who responded ‘yes’ to any question on the *Physical Activity Readiness Questionnaire* (PAR-Q) [37] required medical clearance prior to participating in the trial. Data collection was completed in January 2016.

### Sample size

To date there has been no study investigating the effect of Bikram yoga on parameters of HRV. Therefore, we based our sample size estimate on results derived from studies investigating the effect of aerobic exercise training on HRV in apparently healthy adults [38]. The experimental group was expected to increase the HF component of HRV following the 16-week yoga intervention (568.0 ± 696.0 ms^2^) and the control group was expected to experience no change (205.7 ± 290.5 ms^2^). With a one-sided alpha level of 0.05, at least 56 participants (28 per group) were required to provide 80% power to detect a statistically significant difference between groups. Recruitment was inflated to 68 participants to enable a 15% participant attrition rate.

### Randomization

Participants were randomized via a computer-generated list (www.randomization.com) stratified by sex and age (<50 yr.; ≥50 yr). An investigator not involved in testing or the delivery of the intervention prepared the randomization assignments. Group assignments were delivered to participants in person in sealed envelopes upon the completion of baseline testing.

### Interventions

#### Experimental group

**Fig. 1:**
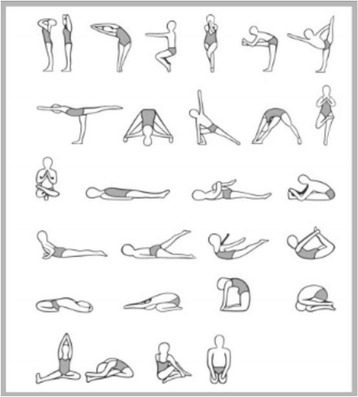
Bikram yoga sequence of *asanas* (left to right, top to bottom)

Participants in the experimental group engaged in 16-weeks of Bikram yoga classes (Fig. 1) at either of two affiliated Bikram yoga studios in Canberra, ACT. Participants were instructed to attend between three to five regularly scheduled classes per week. Certified Bikram yoga teachers instructed all classes using a set instructional dialogue. Classes were 90 min in duration and held in a temperature-controlled room (40 °C, 40% relative humidity). The Bikram yoga practice consisted of 45-50 min of standing *asanas* starting with a deep breathing exercise, and 40-45 min of floor-based *asanas*, including a quick, forceful breathing exercise to finish. All but the last *asana* (spine-twisting) were performed twice. *Savasana* (a restorative, relaxation posture) was performed between *asanas* in the floor series and at the end of class [26].

#### Control group

Participants in the control group were instructed to maintain current lifestyle practices and were not provided any information or instructions about Bikram yoga practice. However, participants were informed during recruitment and screening that the control group participants would be provided a complimentary 10-class pass at one of the participating studios upon completion of the trial.

### Outcome measures

Two trained researchers collected all outcome measures at the University of Canberra, with the exception of hematological data, which were collected and analyzed on a separate day by Capital Pathology, a National Association of Testing Authorities (NATA) certified blood collection center (blinded). Participants were instructed to fast overnight (12 h) and refrain from exercise for 48 h prior to both the two testing session appointment and the blood test appointment. Appointments were rescheduled if these instructions were not followed. Follow up testing was scheduled at the same time of day as baseline testing to minimize the effect of diurnal variation.

#### Heart rate variability outcomes

HRV was measured in a quiet room according to guidelines developed by the Task Force for Pacing and Electrophysiology [14]. After 10 min of supine rest with a regular and calm breathing pattern, a continuous 10-min ECG recording was collected using the SphygmoCor system and HRV software (SphygmoCor, AtCor Medical Pty, Sydney, Australia). From the ECG recording, the following time domain variables were calculated from r-wave to r-wave (RR) intervals: standard deviation of normal-to-normal (NN) intervals (SDNN), root mean square of successive differences between adjacent NN intervals (RMSSD), the proportion of the number of pairs of successive NN intervals that differ by 50 ms divided by the total NN intervals (pNN50) and HRV Triangular Index. Frequency domain variables, including HF and LF power (absolute), LF:HF and total power (TP) were derived from spectral analysis of successive RR intervals. The primary outcome of the present study was the HF spectral power component of HRV (measured in absolute units; ms^2^), while the other HRV measures were collected as secondary outcomes.

#### Hemodynamic outcomes

Resting brachial blood pressure (systolic and diastolic) and heart rate were assessed after lying for 10 min in a supine position using an automatic monitor (M10-IT, Omron Inc., Japan). Augmentation index (AIx) was then measured in this position at the radial artery using the SphygmoCor System (AtCor Medical Pty, Sydney, Australia) and hand-held, high fidelity tonometer (Millar Instruments, Houston, Texas). AIx data was normalized to a heart rate of 75 beats per minute to minimize the effects of confounding variables heart rate and ejection fraction [39].

#### Hematological outcomes

High-sensitivity c-reactive protein (hsCRP), triglycerides, total cholesterol, low-density- and high-density lipoprotein cholesterol (LDL and HDL), total cholesterol to HDL ratio (TC:HDL) and fasting blood glucose were collected and assessed using standard blood collection procedures and assays (coefficients of variation: 2.0% – 4.2%).

#### Anthropometrics and body composition outcomes

Height and weight were measured using a Seca 763 Electronic weighing and measuring scale (Seca, Hamburg, Germany) and BMI was computed from these measures [40]. Waist circumference was measured using a standardized procedure [40]. Body composition was assessed using a Lunar Prodigy Pro™ Dual-energy X-ray Absorptiometry (DXA) scan analyzed with manufacturer software (enCORE™ v 14.1 software, GE Healthcare, Sydney, Australia). DXA scanning was completed in accordance with the University of Canberra’s DXA scanning protocol and has been validated in previous research [41, 42]. Participants were scanned prior to any exercise being completed and wore minimal clothing with all jewelry and metal objects removed. Data from participants too large to fit within the scanning region was estimated using the software estimate function. Percent body fat (%), fat mass (g), lean mass (g), fat mass (g), and bone mineral content (g) were reported for all participants.

### Health status covariates, attendance and adverse events

Weekly status checks administered via phone, email or in person throughout intervention period were used to check for major exercise or diet changes and adverse events in the experimental and control group. A 7-day food diary was also completed in weeks 0 and 17 and analyzed using FoodWorks (version 8, Xyris Software Pty Ltd., Australia) to assess changes in diet. Attendance in the experimental group was recorded electronically upon arrival at each respective studio via an online booking system controlled by the staff member at reception. Attendance was reported as total number of sessions completed. Adverse events were defined as any injury directly attributable to the Bikram yoga intervention. Participants who experienced an adverse event were advised to visit a qualified health care practitioner for assessment and treatment.

### Statistical analyses

Primary analysis was undertaken using intention-to-treat regardless of dropout or level of adherence. Missing data at week 17 was imputed using the last observation carry forward method. Outcomes data is presented as the mean ± standard deviation (SD) with effect size and 95% confidence intervals (CIs). Baseline characteristics were compared using t-tests (continuous variables) and chi square tests (categorical variables). Natural logarithm transformations were applied to variables showing positive skew, and accepted where normality was improved. Mean differences in outcomes between groups at completion were examined using analysis of covariance (ANCOVA) adjusting for the baseline value of the outcome variable. Pearson’s correlation coefficients (*r*) were used to examine baseline associations between the primary outcome and secondary outcomes with *p*-values reported for the hypothesis testing that the correlation was equal to zero. Regression analyses were used to examine the effect of attendance on outcomes. A *p* value less than 0.05 was considered indicative of statistical significance with *p* values less than 0.10 providing weaker evidence of association. Effect sizes are summarised as partial eta-squared statistics. All analyses were carried out using SPSS (IBM**©**, Version 23).

## Results

### Participants

**Fig. 2:**
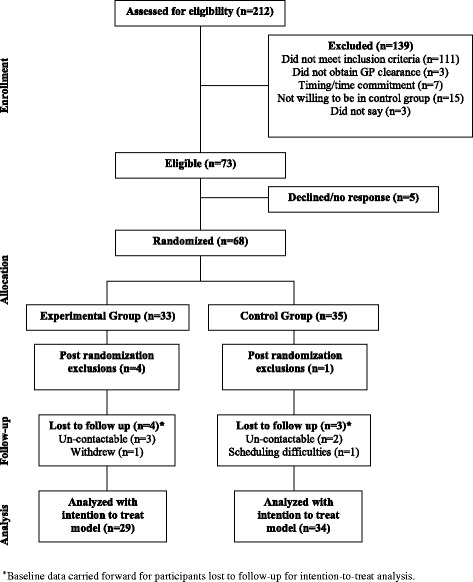
Participant flow

Two-hundred and twelve individuals contacted the principal investigator and were reviewed for eligibility; 139 were deemed ineligible for reasons presented in Fig. 2. Of 73 eligible individuals, 68 provided written informed consent and were randomized to either the experimental or control group. Four participants in the experimental group and one participant from the control group were excluded from analysis post-randomization after re-calculation of the DASS-21 stress scores (inclusion criterion score not met). Further, four participants in the experimental group and three participants in the control group did not return for follow-up testing (week 17).

### Baseline characteristics

At baseline there were no significant differences between groups in any of the descriptive characteristics presented in Table 1. Participants ranged from 19 to 64 years of age. The majority of the cohort (87%) was under 50 years of age (55/63) and 79% of the cohort was comprised of women (50/63). Further, 43% of the cohort (27/63) meet the clinical criteria for obesity according to BMI >30 kg/m^2^. The average DASS-21 stress score for the total cohort indicated a moderate level of stress (23.8 ± 6.0). Nearly 70% of the total cohort had no prior Bikram yoga experience while 90% had engaged in less than five Bikram yoga classes previously.

**Table 1:** Baseline characteristics

Characteristic	Total cohort (*n* = 63)	Yoga group (*n* = 29)	Control group (*n* = 34)
Age (y)	37.2 ± 10.8	38.2 ± 10.1	36.3 ± 11.4
Women (n; %)	50; 79%	23; 79%	27; 79%
Body weight (kg)	88.5 ± 21.0	86.4 ± 21.2	90.4 ± 21.0
Body mass index (BMI) (kg/m^2^)	30.5 ± 6.2	29.9 ± 6.2	30.9 ± 6.3
Obese (BMI 30+) (n; %)	27; 43%	11; 38%	16; 47%
Waist circumference (cm)	94.7 ± 14.7	93.9 ± 14.5	95.3 ± 15.1
Stress score (DASS-21)	23.8 ± 6.0	23.3 ± 6.0	24.3 ± 6.1
Current smoker or quit in last 6 months (n; %)	3; 5%	1; 3%	2; 6%

Continuous data presented as mean ± standard deviation

*Abbreviations*: *DASS* Depression-Anxiety-Stress-Scale (stress categories: 15-18 = mild, 19-25 = moderate, 26-33 = severe, 34+ = extremely severe)

### Health status covariates, attendance and adverse events

There were no significant changes between groups over time in total energy, carbohydrate, protein and fat intake according to the 7-day food diary. Weekly status checks revealed that three participants in the control group engaged in structured exercise programs during the intervention period (i.e. aerobic and/or strength training).

The experimental group attended an average of 27 ± 18 (range: 4 to 79) of a possible 80 classes. Nine participants attended >80% of the minimum three required classes per week (i.e. >38 of 48 classes). Ten participants attended a total of 16 or fewer classes (i.e. less than one class per week) over the intervention period.

Six participants in the experimental group experienced exacerbation of a pre-existing condition during the trial (i.e. back pain, foot pain, knee pain, calf pain, psychological discomfort). All six participants were advised to consult their general practitioner. Three of these participants discontinued the intervention. The other three continued attending some classes when they could, with modifications made to *asanas* if needed. One additional participant discontinued the intervention after a non-intervention related event (sprained ankle). In the control group, one participant reported a meniscus tear incurred by stepping awkwardly.

### Baseline associations with the primary outcome

After logarithmic transformation (Ln) to correct skewed distributions, the Ln HF power component of HRV was found to be positively correlated with all secondary HRV outcomes (all *r* > 0.6, all *p* < 0.001). Further, higher Ln HF power was associated with lower age (*r* = −0.44, *p* < 0.001), resting heart rate (*r* = −0.47, *p* < 0.001), systolic blood pressure (*r* = −0.33, *p* = 0.008), diastolic blood pressure (*r* = −0.27, *p* = 0.031), total cholesterol (*r* = −0.26, *p* = 0.038), LDL cholesterol (*r* = −0.26, *p* = 0.038), and TC:HDL (*r* = −0.33, *p* = 0.008).

### Outcomes

All outcomes are presented in Table 2.

**Table 2:** Summary of between group changes on clinical outcomes

Outcome Measure	Yoga (*n* = 29)		Control (*n* = 34)		Unadjusted Mean difference (95% CI)	*P* (between groups)	Effect Size (partial *η* ^2^)
	Week 0	Week 17	Week 0	Week 17			
Heart Rate Variability							
Ln HF power (absolute)	6.39 ± 1.30	6.27 ± 1.30	5.67 ± 1.46	5.76 ± 1.48	−0.503 (−1.203 – 0.196)	0.912	0.000
Ln LF power (absolute)	6.14 ± 1.17	5.93 ± 1.09	5.66 ± 1.10	5.44 ± 1.19	−0.488 (−1.063 – 0.088)	0.424	0.011
Ln LF:HF ratio	−0.244 ± 0.95	−0.337 ± 0.92	−0.002 ± 1.17	−0.213 ± 1.17	0.124 (−0.406 – 0.653)	0.978	0.000
Ln total power	7.52 ± 1.01	7.33 ± 1.05	7.02 ± 0.94	6.92 ± 1.06	−0.406 (−0.940 – 0.128)	0.755	0.002
pNN50	21.81 ± 21.35	21.46 ± 22.71	18.72 ± 20.74	21.80 ± 24.49	0.338 (−11.566 – 12.243)	0.353	0.014
Ln SDNN	3.99 ± 0.46	3.95 ± 0.48	3.74 ± 0.44	3.77 ± 0.50	−0.183 (−0.429 – 0.063)	0.948	0.000
Ln RMSSD	3.78 ± 0.65	3.80 ± 0.68	3.50 ± 0.68	3.61 ± 0.68	−0.188 (−0.531 – 0.156)	0.942	0.000
Ln Triangular Index	5.91 ± 0.45	5.92 ± 0.43	5.85 ± 0.48	5.83 ± 0.51	−0.098 (−0.333 – 0.138)	0.486	0.008
Hemodynamic measures							
Resting heart rate (bpm)	64.1 ± 8.0	62.9 ± 8.3	65.4 ± 9.1	62.2 ± 8.7	−0.784 (−5.084 – 3.516)	0.248	0.022
Systolic blood pressure (mmHg)	120.3 ± 11.1	119.1 ± 10.3	119.8 ± 9.1	119.6 ± 8.9	0.49 (−4.419 – 5.399)	0.517	0.007
Diastolic blood pressure (mmHg)	74.7 ± 7.9	73.5 ± 8.0	76.1 ± 6.4	74.3 ± 7.2	0.875 (−2.970 – 4.721)	0.769	0.001
Augmentation index (@75 bpm)^a^	16.1 ± 9.8	16.2 ± 8.8	12.9 ± 13.1	13.5 ± 11.3	−2.678 (−7.842 – 2.487)	0.294	0.022
Hematological measures							
Total cholesterol (mmol/L)	5.15 ± 0.84	5.15 ± 0.69	5.03 ± 0.91	4.98 ± 1.11	−0.169 (−0.628 – 0.289)	0.635	0.004
HDL (mmol/L)	1.59 ± 0.52	1.53 ± 0.47	1.40 ± 0.38	1.37 ± 0.34	−0.157 (−0.369 – 0.055)	0.886	0.000
TC/HDL ratio	3.53 ± 1.24	3.65 ± 1.20	3.83 ± 1.25	3.84 ± 1.26	0.187 (−0.435 – 0.808)	0.450	0.010
LDL (mmol/L)^a^	2.94 ± 0.75	3.07 ± 0.71	3.03 ± 0.84	3.02 ± 1.01	−0.045 (−0.481 – 0.390)	0.412	0.011
Triglycerides (mmol/L)	1.27 ± 0.93	1.22 ± 0.64	1.28 ± 0.81	1.28 ± 0.65	0.065 (−0.260 – 0.391)	0.522	0.007
Fasting blood glucose (mmol/L)	5.01 ± 0.58	5.04 ± 0.57	4.94 ± 0.44	4.94 ± 0.52	−0.100 (−0.377 – 0.177)	0.672	0.003
Ln hsCRP	0.64 ± 1.35	0.51 ± 1.27	0.80 ± 1.23	0.78 ± 1.22	0.268 (−0.363 – 0.900)	0.430	0.010
Body Composition^a^							
Body weight (kg)	86.4 ± 21.2	86.2 ± 21.3	90.4 ± 21.0	91.3 ± 21.3	4.328 (−6.279 – 14.935)	0.618	0.004
Body mass index (kg/m^2^)	29.9 ± 6.2	29.8 ± 6.2	30.9 ± 6.3	31.0 ± 6.3	1.186 (−1.977 – 4.349)	0.496	0.008
Waist circumference (cm)	93.9 ± 14.5	92.6 ± 14.7	95.3 ± 15.1	95.0 ± 14.1	2.399 (−4.898 – 9.696)	0.204	0.027
Fat mass (kg)	34.5 ± 14.5	34.4 ± 14.4	38.0 ± 14.0	37.5 ± 13.8	3.141 (−4.066 – 10.348)	0.714	0.002
Lean mass (kg)	48.8 ± 10.4	48.8 ± 10.7	48.3 ± 11.0	48.9 ± 10.8	0.117 (−5.390 – 5.624)	0.126	0.039
Fat-free mass (kg)	51.7 ± 10.9	51.8 ± 11.2	51.4 ± 11.4	52.0 ± 10.9	0.150 (−5.575 – 5.876)	0.147	0.035
Body fat (%)	40.4 ± 9.3	40.4 ± 9.3	43.2 ± 8.7	42.6 ± 8.8	2.188 (−2.442 – 6.819)	0.231	0.024

Data reported as mean (standard deviation). *Abbreviations HF* high frequency, *LF* low frequency, *pNN50* percentage of absolute differences between successive normal RR intervals that exceed 50 ms, *SDNN* standard deviation of the normal-normal interval, *RMSSD* root-mean-square of the successive normal sinus RR interval difference, *HDL* high-density lipoprotein, *TC/HDL* ratio of total cholesterol to HDL, *LDL* low-density lipoprotein, *hsCRP* high sensitivity c-reactive protein

* = *p* < 0.05

^a^ = sample size differs to main data set. DEXA data: yoga group *n* = 28. AIx: baseline yoga group *n* = 22, baseline control group *n* = 30, completion yoga group *n* = 27. LDL: baseline yoga group *n* = 28

#### Heart rate variability

No significant difference in the Ln HF power component of HRV was observed between groups over time (*p* = 0.912, partial *η* ^2^ = 0.000). Further, no group by time interactions were noted in any of the secondary HRV outcomes, including Ln LF power (*p* = 0.424, partial *η* ^2^ = 0.011), Ln LF:HF (*p* = 0.978, partial *η* ^2^ = 0.000), Ln total power (*p* = 0.755, partial *η* ^2^ = 0.002), pNN50 (*p* = 0.353, partial *η* ^2^ = 0.014), Ln SDNN (*p* = 0.948, partial *η* ^2^ = 0.000), Ln RMSSD (*p* = 0.942, partial *η* ^2^ = 0.000) and Ln Triangular Index (*p* = 0.486, partial *η* ^2^ = 0.008).

#### Hemodynamic outcomes

No differences between groups over time were noted for resting heart rate (*p* = 0.248, partial *η* ^2^ = 0.022), systolic blood pressure (*p* = 0.517, partial *η* ^2^ = 0.007), diastolic blood pressure (*p* = 0.769, partial *η* ^2^ = 0.001) or AIx (*p* = 0.294, partial *η* ^2^ = 0.022).

#### Hematological outcomes

No differences between groups over time were found in total cholesterol (*p* = 0.635, partial *η* ^2^ = 0.004), HDL (*p* = 0.886, partial *η* ^2^ = 0.000), TC:HDL (*p* = 0.450, partial *η* ^2^ = 0.010), LDL (*p* = 0.412, partial *η* ^2^ = 0.011), triglycerides (*p* = 0.522, partial *η* ^2^ = 0.007), fasting blood glucose (*p* = 0.672, partial *η* ^2^ = 0.003) or Ln hsCRP (*p* = 0.430, partial *η* ^2^ = 0.010).

#### Anthropometric and body composition outcomes

No differences between groups over time were found in body weight (*p* = 0.618, partial *η* ^2^ = 0.004), BMI (*p* = 0.496, partial *η* ^2^ = 0.008) or waist circumference (*p* = 0.204, partial *η* ^2^ = 0.027). There were also no group by time interactions noted for body fat percentage (*p* = 0.231, partial *η* ^2^ = 0.024), fat mass (*p* = 0.714, partial *η* ^2^ = 0.002), lean body mass (*p* = 0.126, partial *η* ^2^ = 0.039), or fat free mass (*p* = 0.147, partial *η* ^2^ = 0.035). The data of six participants who did not fit comfortably within the scan region were calculated using the software estimate function.

### Effect of adherence on adaptation

ANCOVAs indicated that higher attendance in the experimental group (i.e. number of classes) was associated with significant reductions in diastolic blood pressure (*p* = 0.039, partial *η* ^2^ = 0.154), body fat percentage (*p* = 0.001, partial *η* ^2^ = 0.379), fat mass (*p* = 0.003, partial *η* ^2^ = 0.294) and BMI (*p* = 0.05, partial *η* ^2^ = 0.139). Moreover, weak evidence of association was noted between higher attendance and reduced systolic blood pressure (*p* = 0.072, partial *η* ^2^ = 0.119), body weight (*p* = 0.062, partial *η* ^2^ = 0.128), waist circumference (*p* = 0.072, partial *η* ^2^ = 0.120) and HDL cholesterol (*p* = 0.052, partial *η* ^2^ = 0.137).

## Discussion

The present study investigated the effect of 16-weeks of Bikram yoga on the HF component of HRV, and secondary CVD risk factors in sedentary, stressed adults. Intention-to-treat analysis revealed that the Bikram yoga intervention did not significantly improve the HF power component of HRV or any other CVD risk factors investigated, contrary to our hypotheses. Attendance in the experimental group (*n* = 29) was low, averaging 27 classes, which is 56% of the minimum 48 classes required. Low attendance may have contributed to our null findings. Regression analyses revealed that higher attendance was associated with significant reductions in diastolic blood pressure, body fat percentage, fat mass, and BMI. Continued investigation into adherence to Bikram yoga interventions may reveal further information on the dose-response effect of Bikram yoga on CVD risk factors.

Our findings for HRV outcomes are supported by a recent systematic review [25], which concluded that current evidence cannot describe a definitive effect of chronic *hatha* yoga practice on measures of HRV, including HF power HRV, LF power HRV, LF:HF, TP, SDNN, and RMSSD. The HF component of HRV can be increased acutely by a reduced respiration rate, which likely contributed to the positive findings noted in some acute trials [25, 43]. The present study suggests that there is no chronic increase in vagal tone (relaxation response) in response to the intervention, however previous research suggests that Bikram and non-Bikram yoga interventions do reduce psychological stress [16, 28] and reduce cortisol reactivity to stress [27]. Heart rate variability can also be increased from via exercise-induced adaptation of the cardiovascular system. Research to date indicates that Bikram yoga does not elicit a strong enough cardiovascular training load in sedentary, apparently healthy adults to improve maximal oxygen update [29, 44]. The null findings in the present study may be due to the lack of chronic adaptation of the cardiac muscle (i.e. increased stroke volume) required for adaptation in HRV despite past evidence for reduced psychological stress from Bikram yoga intervention. Preliminary data show that Bikram yoga elicits an average, in-session metabolic response equivalent to that of walking [45, 46] which may, in a higher risk, less fit population, lead to increased HRV via adaptation in cardiac muscle and stroke volume after a Bikram yoga intervention. Interestingly, a study examining integrated *hatha* yoga (ethical and spiritual components) versus *hatha* yoga (as exercise only) reports that only those in the integrated group experienced significant decreases in physiological measures of stress (i.e. salivary cortisol) [47]. This finding suggests that the framework of *hatha* yoga RCTs, including the reasons for which participants initially register, may influence adaptations of physiological stress markers.

There was a null effect of Bikram yoga on secondary hemodynamic outcomes in the present study. Our findings in this cohort of normotensive adults (Table 2) support those of previous studies (controlled and uncontrolled), which report unchanged blood pressure and resting heart rate in normotensive adults after 8 weeks of Bikram or non-Bikram *hatha* yoga performed one to three sessions per week [24, 28, 29, 44]. Interestingly, a cross-sectional study reported that resting blood pressure of long-term Bikram yoga practitioners (1 year) was lower than the general United States population indicating that this practice may have a beneficial effect on hemodynamic health [48]. Moreover, longer (5-6 months) *hatha* yoga RCTs report significant improvements in blood pressure in coronary artery disease patients and pre-hypertensive individuals living with HIV infection [49–51]. A previous study in healthy young adults has also noted within group reductions in arterial stiffness measured at the carotid artery after 8 weeks of Bikram yoga intervention [29]. The significant association between adherence and diastolic blood pressure (*p* = 0.039, partial *η* ^2^ = 0.154), and weaker evidence of association in systolic blood pressure (*p* = 0.072, partial *η* ^2^ = 0.119) in the present study indicate that there may be a dose-response effect for blood pressure changes in relation to Bikram yoga. Considering both the relationship between adherence and adaptation, and the potential vascular benefits of heat therapy shown in several studies [31, 52, 53], an RCT in a pre-hypertensive or hypertensive cohort, with adequate adherence to intervention, may elicit Bikram yoga intervention-related changes in systolic and diastolic blood pressure.

No significant changes were found for hematological CVD risk factors. An 8-week, uncontrolled Bikram trial also reported no change to fasting blood glucose in adults, although improvements in glucose tolerance were noted in obese adults [29]. Contrary to the present results, however, an uncontrolled 8-week Bikram yoga trial reported significant within group decreases in TC and LDL in older adults, and significant within group decreases in TC and HDL in young adults [29]. The current trial also provides weak evidence for reduced HDL with higher attendance (*p* = 0.052, partial *η* ^2^ = 0.137). This is an unexpected HDL change in response to exercise, however, HDL levels were 1.53 ± 0.47 mmol/L at completion, which is still well above recommended levels for a healthy blood lipid profile (>1.0 mmol/L). The same 8-week study also reports that plasma insulin and insulin resistance via homeostatic model assessment (HOMA-IR) improved significantly (within group) in older adults [29]. Further, RCTs longer than 8 weeks suggest that *hatha* yoga can improve blood lipids, blood glucose and hemoglobin A1c in unhealthy populations [49, 50, 54]. There was no adaptation in hsCRP in the present study. This finding is contrary to a 4-week *hatha* yoga RCT in apparently healthy, male railway engine drivers, and an 8-week *hatha* yoga RCT in heart failure patients [55, 56]. Although adherence did not appear to be associated with blood measure outcomes, it is possible that a higher risk cohort with elevated CVD risk factor blood measures at baseline would experience significant reductions in response to an appropriate volume of Bikram yoga training.

There was no significant adaptation of body weight, body composition, waist circumference or BMI in the present study. An RCT reported a trend in reduced body fat after 8-weeks of Bikram yoga, although no diet data was recorded during that 8-week trial [44]. Preliminary energy expenditure data from two studies indicates that Bikram yoga elicits a higher metabolic equivalent (MET) energy expenditure level compared to other forms of *hatha* yoga, and that it can be compared energetically to walking up to 3.7 METS [45, 46, 57]. Both studies include data from experienced practitioners, who may have higher exertion and energy expenditure rates during class than that of the current, less experienced, less active practitioners. Yoga has been associated with attenuated weight gain in healthy adults over a 10-year period [58]. In the current trial, more regular attendance was associated with reductions in body fat percentage (*p* = 0.001, partial *η* ^2^ = 0.379), fat mass (*p* = 0.003, partial *η* ^2^ = 0.294) and BMI (*p* = 0.05, partial *η* ^2^ = 0.139). These data suggest that Bikram yoga may serve as an effective weight maintenance tool and, with an appropriate training volume, a tool for body fat reduction.

A major strength of this study was its rigorous RCT design [59], which is currently lacking in the investigation of Bikram yoga. A major limitation to the study was low attendance with participants attending on average 1.7 classes per week compared to the minimum requirement of three classes per week. Poor attendance could be due to the duration of the 16-week trial or the time demand of the 90-min classes. Another limitation to the study is that the cohort may have been too low-risk to see adaptation in certain measures including blood pressure, blood lipids and hsCRP. Further, data was collected without considering the timing in relation to menstrual cycle. In this predominantly female cohort, that oversight may have affected some outcome measures. Some participants experienced discomfort relating to pre-existing musculoskeletal conditions during the study, which would not be uncommon when starting an exercise program after being sedentary for some period. The intervention may have exacerbated this discomfort for some participants due to the group exercise setting. To reduce intervention-related discomfort, future study design could include one pre-intervention yoga class to better familiarize participants with the protocol and to ensure that they are given specific, individual feedback for performing the *asanas* in a way that minimizes discomfort.

Future research should explore the effects of Bikram yoga in unhealthy populations to better examine the effects of this form of heated *hatha* yoga on CVD risk factors. However, in light of the association of adherence on the outcomes demonstrated in this study, more acute and intervention-based research is required to determine a suitable, minimum volume of Bikram yoga in order to encourage an adequate level of adherence that elicits physiological change in various outcomes. Furthermore, cross-sectional and longitudinal data on long term practitioners could lend valuable insight into the characteristics of a Bikram yoga practitioner, including motivation to start and continue a Bikram yoga practice, as well as perceived barriers to continue. Determining how to better facilitate adherence to Bikram yoga interventions is an important consideration moving forward, especially considering that a stressed cohort with little yoga experience potentially faces more barriers to initiating a yoga practice than an individual initiating a yoga practice independent of the RCT framework.

## Conclusion

In summary, a 16-week Bikram yoga program did not increase the HF power component of HRV or any other CVD risk factors investigated. Low adherence likely contributed to the null effects. Regression analyses indicated that higher attendance was significantly associated with reductions in diastolic blood pressure, body fat, fat mass and BMI, and weaker associations were noted in systolic blood pressure, body weight and waist circumference. Future studies are required to address barriers to adherence and elucidate the dose-response effects of Bikram yoga practice.

## Abbreviations

ACT: Australian capital territory
AIx: Augmentation index
ANCOVA: Analysis of covariance
ANS: Autonomic nervous system
BMI: Body mass index
CI: Confidence interval
CVD: Cardiovascular disease
DASS-21: 21-item depression-anxiety-stress scale
DXA: Dual-energy x-ray absorptiometry
ECG: Electrocardiogram
HF: High frequency
HDL: High-density lipoprotein
HPA: Hypothalamic-pituitary-adrenal
HRV: Heart rate variability
hsCRP: High-sensitivity c-reactive protein
LF: Low frequency
LDL: Low-density lipoprotein
Ln: Natural logarithm
MET: Metabolic equivalent
NATA: National Association of Testing Authorities
NN: Normal-to-normal
PAR-Q: Physical activity readiness questionnaire
pNN50: proportion of the number of pairs of successive NN intervals that differ by 50 ms divided by the total NN intervals
RCT: Randomized controlled trial
RMSSD: Root mean square of successive differences between adjacent NN intervals
RR: r-wave to r-wave
SD: Standard deviation
SDNN: Standard deviation of NN intervals
SNS: Sympathetic nervous system
SPSS: Statistical package for the social sciences
TC: Total cholesterol
TP: Total power
